# Association of Statin Use with Reduced Primary Liver Cancer Risk, Independent of Age and Cirrhosis Protection in MASLD

**DOI:** 10.3390/cancers18071132

**Published:** 2026-04-01

**Authors:** Georgia Sofia Karachaliou, Amy M. Perkins, Chad Dorn, Ruth M. Reeves, Timothy Arnold, Mustafa R. Bashir, Jimmy T. Efird, Manal F. Abdelmalek, Anna Mae Diehl, Ayako Suzuki

**Affiliations:** 1 Division of Gastroenterology, Duke University Medical Center, Durham, NC 27710, USA; georgiasofia.karachaliou@duke.edu (G.S.K.); annamae.diehl@duke.edu (A.M.D.); 2 Department of Biostatistics, Vanderbilt University Medical Center, Nashville, TN 37203, USA; amy.perkins@vumc.org; 3 Geriatrics Research Education and Clinical Center (GRECC), VA Tennessee Valley Healthcare System, Nashville, TN 37212, USA; 4 Health Services Research, VA Tennessee Valley Healthcare System, Nashville, TN 37212, USA; chad.a.dorn@vumc.org (C.D.); ruth.reeves@vumc.org (R.M.R.); 5 Department of Biomedical Informatics, Vanderbilt University Medical Center, Nashville, TN 37203, USA; 6 Human Systems Integration, Office of Clinical Informatics, Office of Health Informatics, Digital Health Office, Veterans Health Administration, Washington, DC 20240, USA; timothy.arnold4@va.gov; 7 College of Pharmacy, University of Michigan, Ann Arbor, MI 48109, USA; 8 Department of Radiology, Duke University Medical Center, Durham, NC 27710, USA; mustafa.bashir@duke.edu; 9 Center for Advanced Resonance Developmental, Duke University, Durham, NC 27710, USA; 10 VA Cooperative Studies Program, Coordinating Center, Boston, MA 02130, USA; jimmy.efird@va.gov; 11 School of Medicine, Case Western Reserve University, Cleveland, OH 44106, USA; 12 Division of Gastroenterology and Hepatology, Mayo Clinic, Rochester, MN 55905, USA; abdelmalek.manal@mayo.edu; 13 Division of Gastroenterology, Durham VA Medical Center, Durham, NC 27710, USA

**Keywords:** primary liver cancer, metabolic-dysfunction-associated steatotic liver disease, nonalcoholic fatty liver disease, electronic health records, HMG-CoA reductase inhibitors

## Abstract

**Background:** Statins have been associated with a reduced risk of primary liver cancer (PLC), primarily hepatocellular carcinoma (HCC). However, the optimal use for effective protection and whether benefits vary by patient characteristics remain unclear. We evaluated the association between statin use and PLC risk in metabolic-dysfunction-associated steatotic liver disease (MASLD), considering cumulative exposure and potential effect modifiers. **Methods:** We conducted a retrospective cohort study using the Veteran Affairs electronic health records. Patients with chronically elevated liver enzymes and metabolic dysfunction, without other chronic liver diseases, were identified between 2007 and 2009 and followed through 2019 for incident PLC. Statin exposure was assessed at baseline and during the follow-up, with dose standardization by LDL-lowering potency (simvastatin-equivalent units). Time to PLC was analyzed using Cox models adjusted for covariates, considering potential interactions. **Results:** Among 329,577 patients (92% male; median age 62 years), 0.82% developed PLC (median follow-up of 9.7 years). Baseline statin use showed a significantly lower PLC risk (adjusted hazard ratio 0.64; 95% CI, 0.57–0.71; *p* < 0.0001). No significant interaction was observed with age, sex, metabolic syndrome, or cirrhosis. Higher cumulative statin exposure demonstrated a dose-dependent risk reduction, remaining significant at simvastatin-equivalent doses > 15,561 mg annually after accounting for incident cirrhosis. Atorvastatin/rosuvastatin use provided comparable protection, despite different lipophilicity, and demonstrated stronger effects than others. **Conclusions:** In MASLD, statin therapy was associated with a dose-dependent PLC risk reduction. High-intensity therapy (simvastatin-equivalent > 40 mg daily) conferred substantial protection regardless of age, sex, insulin resistance, or cirrhosis, supporting a potential statin-based PLC chemoprevention in MASLD.

## Simple Summary

This 10-year retrospective cohort study using a large unified electronic health record system evaluated the association between statin exposure and incident primary liver cancer (PLC). After accounting for potential effect modification by age, sex, metabolic syndrome, and cirrhosis, statin use was associated with a dose-dependent reduction in PLC risk. Analyses incorporating cumulative statin exposure indicated that high-intensity therapy (simvastatin-equivalent > 40 mg daily) was associated with lower PLC risk, independent of age, insulin resistance, or cirrhosis status. Assessment of effect modification by sex was limited by the predominantly male cohort. This association remained significant after adjustment for incident cirrhosis, suggesting a potential effect beyond cirrhosis prevention. These findings suggest a possible chemopreventive role of statins in MASLD-related PLC and warrant further investigation.

## 1. Introduction

Liver cancer represents a significant global health burden, ranking as the sixth most diagnosed cancer and the third leading cause of cancer-related death worldwide [1]. In the U.S., incidence rates have more than tripled since 1980, even as overall cancer mortality has declined [2]. While chronic hepatitis C and B and excessive alcohol use remain well-established drivers of hepatocellular carcinoma (HCC) [3], the growing burden of obesity, diabetes, and metabolic-dysfunction-associated steatotic liver disease (MASLD) is increasingly contributing to disease incidence [3,4]. Importantly, MASLD has emerged as the fastest-growing underlying etiology of HCC among liver transplant candidates and is expected to become the leading cause of HCC in the U.S. [5]. Unlike other chronic liver diseases, HCC in MASLD often occurs without cirrhosis; approximately 35% of MASLD-related HCC cases arise in non-cirrhotic liver [6]. Additionally, an increased risk of cholangiocarcinoma (CCA) has been linked to metabolic conditions and MASLD [7,8], although findings in the literature remain inconsistent [9]. Given the limited availability of effective MASLD treatment, there is an urgent need to identify preventive strategies to reduce liver cancer risk in this population.

Statins, 3-hydroxy-3-methylglutaryl coenzyme A (HMG-CoA) inhibitors, have demonstrated protective effects against various liver-related outcomes in chronic liver diseases, including MASLD. While multiple hepatoprotective mechanisms have been proposed, it remains unclear whether reduced liver cancer risk is primarily mediated through protection against cirrhosis, through independent pathways, or by modulation of hepatic cholesterol metabolism. In cardiovascular prevention, statins are equally effective across sex [10], but whether their protective effects against liver cancer are similarly consistent or modified by sex, age, or reproductive status remains uncertain. Given the known sexual dimorphism in MASLD pathogenesis [11] and the documented sex-specific transcriptional responses to statins [12], it is important to evaluate potential disparities in chemopreventive effects to inform individualized strategies.

In our recent retrospective cohort study of patients with chronic liver enzyme elevation associated with metabolic dysfunction, statins showed a dose-dependent protective effect against cirrhosis when standardized by low density lipoprotein (LDL)-lowering intensity across formulations [13]. This effect was more pronounced in older individuals, consistent with emerging evidence implicating cellular senescence in MASLD progression and suggesting a potential role of statins in modulating senescence-related pathways [14,15]. These findings highlight statins’ broader hepatoprotective potential beyond lipid lowering. However, whether statins confer similar dose- and age-dependent protection against primary liver cancer remains unclear. In the current study, we addressed the optimization of individualized strategies for liver cancer chemoprevention.

## 2. Methods

*Study Design and Data Source:* This retrospective cohort study utilized electronic health records (EHR) data from the Veterans Affairs (VA) Corporate Data Warehouse (CDW), accessed through the Veterans Health Administration (VHA) Informatics and Computing Infrastructure (VINCI).

We defined a cohort of patients with chronic liver enzyme elevation accompanied by metabolic features at baseline from 1 January 2007 through 31 December 2009. The cohort was followed for up to 10 years to identify incident PLC.

Our specific aims were to evaluate the association between baseline and follow-up statin exposure and incident PLC, accounting for potential effect modification by age, sex, baseline metabolic syndrome and prevalent cirrhosis as well as variation by statin formulation after standardizing dose by LDL-lowering intensity.

This study was approved by the Institutional Review Board of the Durham VA (Pro#02289), Duke University (Pro#00106275), and Nashville VA (Pro#1602086) as well as the U.S. Army Medical Research & Development Command, Office of Human Research Oversight (HRPO Log Number E01745.1b). The study was granted a Category 4 exemption for secondary research not requiring informed consent and was conducted in accordance with the Declaration of Helsinki.

*Study Population:*

**Figure 1:**
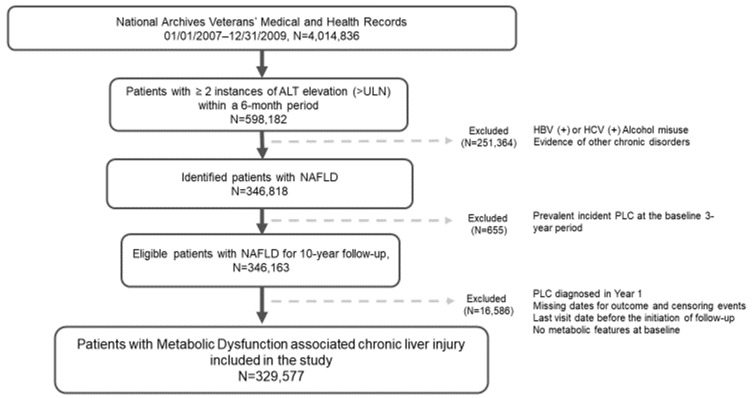
Algorithm used to define the study cohort.

A validated EHR algorithm for nonalcoholic fatty liver disease (NAFLD) [16] was modified to incorporate metabolic dysfunction, allowing for the identification of patients with MASLD. The algorithm was first applied to identify patients during the three-year baseline period with increased alanine aminotransferase (ALT), defined as ≥40 IU/mL for men and ≥31 IU/mL for women, occurring on two or more occasions at least 6 months apart within any 2-year span. Patients with other chronic liver diseases or excess alcohol use were excluded. The remaining cohort was evaluated for coexisting metabolic features at baseline to define the MASLD cohort. Metabolic features used to identify metabolic dysfunction included obesity, type 2 diabetes mellitus, hypertension, and hyperlipidemia, based on International Classification of Diseases [ICD]-9 codes. Patients without any metabolic features at baseline were excluded. The detailed algorithm and exclusion criteria are summarized in Figure 1.

ALT: alanine aminotransferase; UNL: upper limit normal; HBV: hepatitis B virus; HCV: hepatitis C virus; PLC: primary liver cancer; MASLD: metabolic-dysfunction-associated chronic liver disease.

Using the validated electronic health records (EHR) algorithm [16], patients with chronic liver enzyme elevation were identified during the baseline period (1 January 2007–31 December 2009). The following exclusion criteria were applied: (1) active hepatitis B infection (defined as positive hepatitis B surface antigen [HBsAg] or detectable HBV DNA) or active hepatitis C infection (defined as positive HCV antibody and detectable HCV RNA), (2) other chronic liver diseases or alcohol use disorder, identified using ICD-9 codes and a positive Alcohol Use Disorders Identification Test-Consumption [AUDIT-C] screen, (3) prevalent PLC at baseline, (4) diagnosis of PLC within the first year of follow-up, (5) last recorded visit date or death occurring before the start of follow-up (1 January 2010), (6) missing outcome or censoring event dates, and (7) absence of metabolic dysfunction at baseline. AUDIT-C was considered positive for alcohol misuse when the score was 4 for men and 3 for women. After applying these exclusion criteria, the final cohort included 329,577 patients with MASLD, who were followed for up to ten years for incident PLC.

*Study Outcome:* The outcome was incident primary liver cancer, identified using ICD codes from outpatient and inpatient encounters during the follow-up. ICD-9 codes (155.0, 155.1, 155.2, 156.9, 230.8) were used through 30 September 2015, and ICD-10 codes (C22.0, C22.1, C22.7, C22.8, C22.9, C24.8, C24.9) were used from 01 October 2015, when ICD-10 codes were adopted by the VHA. Event dates were based on the coded diagnosis date. The ICD code selection was intentionally inclusive to capture cases of CCA, both intrahepatic as well as others, occurring in the context of MASLD.

As a secondary analysis, we also examined cancer-type-specific cumulative incidence, classifying events by ICD codes, and assessed statin-associated protection across cancer subtypes (see Supplementary Material for details).

*Study Exposures:* Three statin exposure variables were defined as per our previous analysis [13]: (1) baseline statin use, (2) cumulative duration of statin use during the follow-up period, and (3) standardized cumulative statin dose during the follow-up period.

Baseline statin exposure was defined dichotomously (yes/no) based on any statin use at baseline, irrespective of formulation, dose, or duration. Cumulative duration of statin use (in days) was determined on an annual basis using days of supply from both outpatient prescriptions and inpatient dispensing records [13].

Cumulative statin dose was estimated from the prescribed daily dose and days of supply, standardized across formulations according to LDL-cholesterol-lowering potency. Using a published conversion table [17], doses were expressed in simvastatin-equivalent units (Supplementary Table S1) and multiplied by days of supply to derive annual cumulative dose. Both cumulative duration and dose were capped at 365 days per year, with excess carried forward to subsequent years.

*Other Variables Analyzed:* Demographic data, including age (as of 31 December 2009), sex, race (the NIH-defined five race categories plus unknown), and ethnicity (Hispanic or Latino vs. non-Hispanic or Latino), were retrieved from the CDW during the baseline period.

Baseline comorbidities, including type 2 diabetes mellitus, hypertension, hyperlipidemia, and coronary artery disease, were defined using ICD-9 codes within the Observational Medical Outcomes Partnership (OMOP) data structure [18,19,20]. Body mass index (BMI) values nearest to 31 December 2009 were extracted. Baseline exposure to potential confounding comedications (i.e., metformin [21,22,23], beta-blockers [24,25], angiotensin-converting enzyme (ACE) inhibitor/angiotensin receptor blockers (ARBs) [23,26,27], aspirin/NSAIDs [28,29,30] and vitamin D supplements [31,32]) was ascertained from prescription records. The FIB-4 score was determined at baseline to account for underlying hepatic fibrosis.

Time-dependent covariables, including median BMI, diagnoses of type 2 diabetes mellitus, hypertension, and hyperlipidemia (defined using ICD-9/10 codes per the OMOP structure) [18,19,20], as well as the above comedication use, were also created for each follow-up year.

Furthermore, metabolic syndrome, defined as the presence of three or more metabolic features (yes or no) at baseline and during the follow-up, was used as a proxy for insulin resistance [33]. This variable supported the evaluation of a specific hypothesis: that statins may offer greater protective effects among individuals with signs of senescence—either older adults or younger individuals with insulin resistance, which can promote premature senescence [34].

*Censoring Events:* During follow-up, patients were censored at the earliest occurrence of: (1) incident chronic liver disease (defined by ICD-9/10 codes); (2) new or active hepatitis B or C infection (HBsAg positivity or detectable HBV DNA; detectable HCV RNA); (3) alcohol misuse (positive AUDIT-C score [35] or relevant ICD-9/10 codes); (4) loss to follow-up (death or last recorded visit); or (5) end of follow-up (31 December 2019) [13].

*Statistical Methods:* Continuous variables are reported as median (interquartile range [IQR]) and categorical variables as percentages. Baseline characteristics were compared by statin use (yes vs. no) using Mann–Whitney’s U or chi-square tests, as appropriate. Ten-year cumulative incidence of primary liver cancer (PLC), with 95% confidence intervals (CIs), was estimated for the overall cohort and stratified by age (≤65 vs. >65 years) and sex; age 65 was selected based on the median age at PLC diagnosis [36]. Incidence was calculated as the proportion of events over 10 years, without accounting for censoring. Analyses were also performed for specific cancer subtypes (e.g., HCC, CCA) (see Supplementary Material). Confidence intervals were computed using the *Hmisc* package in R.

Missing data (Supplementary Table S2) were addressed using multivariate imputation by chained equations with fully conditional specification (R package *mice*) [37], generating 20 imputed datasets [37]. A Bayesian pattern-mixture missingness approach was conducted to validate that no meaningful departures existed form the Missing at Random (MAR) assumption [38].

Cox’s proportional hazards (PH) models were used to evaluate time to incident primary liver cancer (PLC), with hazard ratios (HRs) estimating relative risk. Six models were specified with sequential adjustment: (1) unadjusted; (2) demographics; (3) baseline comorbidities; (4) baseline comedications; and (5–6) either baseline FIB-4 score or cirrhosis. Patient age was categorized into quartiles (≤53, 54–62, 63–68, ≥69 years) to facilitate assessment of interaction with statin use. Nonlinear associations for continuous covariates were modeled using restricted cubic splines with three knots. Estimates were pooled across 20 multiply-imputed datasets using the *Hmisc* package in R [39]. Pre-specified subgroup analyses were conducted by age (≤65 vs. >65 years) and sex. Due to limited sample size, adjusted models included a reduced set of covariates (age, race/ethnicity [non-Hispanic White, Hispanic, others], baseline metabolic syndrome, with and without cirrhosis). Interaction terms were incorporated to evaluate effect modification by age, metabolic syndrome, and cirrhosis on the association between baseline statin use and incident PLC. Of note is the fact that sex was not included in any interaction terms in the models owing to the limited number of females in the cohort and was analyzed only in the subgroup analysis.

We also assessed the effect of specific statin formulations (i.e., atorvastatin alone, rosuvastatin alone, and simvastatin alone at baseline) compared with no statin use at baseline after adjusting for baseline covariates.

Time-dependent Cox’s PH models were additionally used to examine the associations of cumulative statin dose and duration during follow-up with incident primary liver cancer (PLC). These models included adjustment for both baseline and time-varying covariates. Cumulative statin dose (mg/year) was categorized into four groups, with non-users as the reference and tertiles among users (1–6960, 6961–15,560, and ≥15,561 mg/year). Cumulative duration (days/year) was similarly categorized, with non-users as the reference and three intervals among users (1–122, 123–244, and ≥245 days/year). To ensure appropriate temporal ordering, time-dependent covariates were lagged by one year, such that exposure history up to the prior year informed risk in the subsequent interval. Interaction terms were included to evaluate effect modification by age, metabolic syndrome, and cirrhosis.

Statistical analyses were conducted using the R [40] software version 4.4.1, while graphs were generated with the R package *ggplot2* [41].

## 3. Results

### 3.1. Clinical Characteristics of the Study Population

Table 1 summarizes the clinical characteristics of the overall cohort and by baseline statin treatment (yes or no). Most patients were male (92%), White (78%), and non-Hispanic or Latino (90%), with a median age of 62 years [IQR: 53, 68] and median BMI of 31.6 [IQR: 28.2, 35.6]. At baseline, 79% had ≥3 cardiometabolic features, and 66% were on statin therapy. Compared with non-users, statin users had a higher prevalence of metabolic syndrome and, individually diabetes mellitus, hypertension, hyperlipidemia, and coronary artery disease as well as a higher FIB-4 score and were prescribed greater use of relevant comedications (i.e., metformin, ACE inhibitors, beta-blockers).

Frequency of specific statin formulations used at baseline are summarized in Supplementary Table S3.

### 3.2. Cumulative Incidence of PLC During the Follow-Up and Variations by Age, Sex, and Baseline Cirrhosis Diagnosis

Among 329,577 patients with MASLD at risk, the median follow-up was 9.7 years [IQR:4.1–10.0], with 2717 incident PLC cases (0.82%). Table 2 summarizes the cumulative incidence in the overall cohort and stratified by baseline cirrhosis, age and sex.

As expected, PLC incidence was approximately ten times higher among those with baseline cirrhosis. Men had higher PLC incidence than women in both cirrhosis and non-cirrhosis groups, with a more pronounced sex difference among those without cirrhosis (Table 2). Among men without cirrhosis, incidence was higher in those >65 years, while no clear age difference was observed among cirrhotic men. Women had fewer PLC cases overall, and older women showed a higher incidence, though 95% CIs were wide, which was attributable to the smaller sample size.

Cancer-specific cumulative incidence was also computed, applying an algorithm detailed in Supplementary Material. Cancer-specific cumulative incidence was 0.52% (95%CI: [0.49%, 0.54%]) for HCC, 0.07% (95%CI: [0.06%, 0.08%]) for CCA, 0.04% (95%CI: [0.03%, 0.05%]) for intrahepatic CCA, 0.04% (95%CI: [0.03%, 0.04%]) for both HCC/CCC diagnoses (0.03% 95% CI [0.02%, 0.03%] for both HCC/intrahepatic CCA), and 0.2% (95%CI: [0.18%, 0.21%]) for unspecified liver cancer (Supplementary Table S4).

### 3.3. Unadjusted and Adjusted Association Between Incident PLC and Baseline Statin Use in the Overall Population, Stratified by Age and Sex

Table 3 summarizes the Cox PH models assessing the association between baseline statin use and incident PLC. In the unadjusted model (Model 1), baseline statin use was associated with a higher PLC risk (HR [95%CI]:1.20 [1.08, 1.33], *p* = 0.0004). However, after adjusting for demographic characteristics, this association reversed (see Model 2, HR [95%CI]: 0.88 [0.80, 0.98], *p* = 0.018). Adding cardiometabolic risk factors (Model 3) slightly strengthened the protective effect (HR [95%CI]: 0.81 [0.73, 0.90], *p* < 0.0001), and further adjustment for comedications (Model 4) enhanced it (HR [95%CI]: 0.64 [0.57, 0.71], *p* < 0.0001), suggesting confounding by relevant medications. Adjusting for baseline FIB-4 (Model 5) or incident cirrhosis (Model 6) did not alter the effect size (HRs 0.64 and 0.66, respectively).

The table shows hazard ratios and 95% confidence intervals for baseline statin use (yes vs. no), with *p*-values (Wald tests) from six models. Baseline covariates were incrementally added as follows: Model 1, unadjusted; Model 2, adjusted for age, sex, and race/ethnicity; Model 3, Model 2 plus baseline cardiometabolic factors (obesity, diabetes mellitus, hypertension, hyperlipidemia, and coronary artery disease); Model 4, Model 3 plus comedications of interest (see Section 2); Model 5, Model 4 plus baseline FIB-4 score; and Model 6, Model 4 plus baseline cirrhosis diagnosis. Age was categorized into quartiles (≤53, 54–62, 63–68, and ≥69 years). Due to concerns regarding collinearity and potential endogeneity, subsequent analyses used fully adjusted models including cirrhosis instead of the FIB-4 score.

Analyses stratified by sex and age (≤65 vs. >65 years, Supplementary Table S5) showed significant protective effects only in men, likely attributable to limited statistical power in the female subgroup. The protective effect appeared slightly stronger in older individuals, although CIs were wide.

Furthermore, we investigated the effects of specific statin formulations at baseline compared with no statin use using the fully adjusted model, including baseline cirrhosis (Model 6). Atorvastatin and rosuvastatin demonstrated the strongest protective associations (HR 0.47 [0.23–0.95], *p* = 0.04; HR 0.49 [0.33–0.74], *p* = 0.0007, respectively), while simvastatin showed a more modest effect (HR 0.68 [0.60–0.76], *p* < 0.001). Other statin formulations showed no significant association (HR 0.88 [0.63–1.23], *p* = 0.46).

The cancer-type-specific analysis revealed that the effect of baseline statin use was consistent for HCC and CCA; however, statistical power was limited owing to low frequency of cancer-type-specific events, and the association reached statistical significance only for HCC (Supplementary Table S6).

### 3.4. Association Between Statin Use During the Follow-Up and Incident PLC: Effect Modification by Age and Metabolic Syndrome

Table 4 summarizes HRs and 95% CIs for cumulative statin dose and duration during the follow-up based on the Cox PH models adjusted for baseline and time-dependent covariates.

The models were developed to evaluate the association between cumulative statin exposure during the follow-up period and incident PLC, adjusting for baseline and time-dependent covariates. Model 4 included age, sex, race/ethnicity, cardiometabolic comorbidities (obesity, diabetes mellitus, hypertension, hyperlipidemia, and coronary artery disease), comedications of interest (see Section 2), baseline statin use, and time-dependent covariates (cardiometabolic factors and comedications). Model 6 included all covariates in Model 4 plus baseline cirrhosis diagnosis and incident cirrhosis diagnosis as a time-dependent variable.

**Figure 2:**
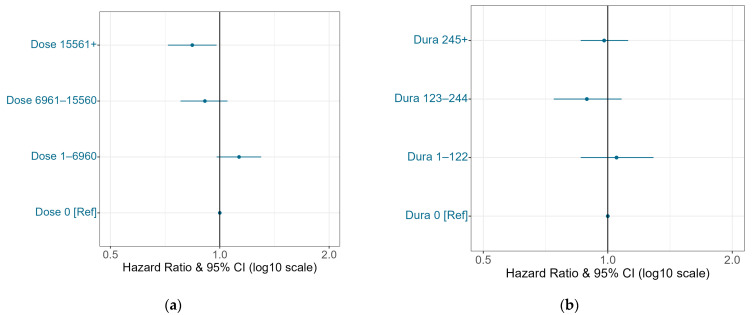
(**a**) Adjusted association between cumulative statin dose and incident primary liver cancer over 10-year follow-up. **Ref**: referent; **CI**: confidence interval. Circles and whiskers represent adjusted hazard ratios (HRs) and 95% CIs on a logarithmic scale for categories of cumulative statin dose, with no statin use as the referent. Estimates were derived from a Cox PH model, including both baseline and time-dependent covariates, including incident cirrhosis. No significant interactions were observed between statin dose and age, metabolic syndrome, or cirrhosis. (**b**) Adjusted association between cumulative statin use duration and incident primary liver cancer over 10-year follow-up. **Ref**: referent; **CI**: confidence interval. Circles and whiskers represent adjusted hazard ratios (HRs) and 95% CIs on a logarithmic scale for categories of cumulative statin use duration, with no statin use as the referent. Estimates were derived from a Cox PH model, including both baseline and time-dependent covariates, including incident cirrhosis. No significant interactions were observed between statin duration and age, metabolic syndrome, or cirrhosis.

A dose-dependent protective association was observed between cumulative statin dose and incident PLC, with significant protection at annual doses ≥ 6961 mg. When incident cirrhosis was included in the model, the protective effect was slightly attenuated (by 0.05–0.07), and significance remained only for annual doses ≥ 15,561 mg (Figure 2a). No significant association was found for cumulative statin use duration (Figure 2b). No significant effect modification was observed by age, metabolic syndrome (two-way), age and metabolic syndrome (three-way), or cirrhotic status (Supplemental Table S7).

## 4. Discussion

This ten-year retrospective cohort study in MASLD using a large EHR dataset yielded several novel findings [13]. In this predominately male cohort, crude cumulative PLC incidence was 0.82% over a median follow-up of 9.7 years, with risk influenced by age, sex, and cirrhosis status. Like in cirrhosis [13], statins showed dose-dependent protection against PLC when standardized by LDL-lowering intensity across formulations. However, in contrast to cirrhosis, this protection was not age dependent.

Metabolic syndrome, as a proxy for insulin resistance, did not modify the effect of statins, alone or in combination with age. After dose standardization, atorvastatin and rosuvastatin showed comparable protection against PLC despite differences in lipophilicity, whereas simvastatin demonstrated a more modest effect. Including both baseline cirrhosis and incident cirrhosis in the model resulted in minimal effect attenuation, suggesting that statins may exert hepatoprotection through multiple mechanisms, (e.g., kinase signaling [42,43]). A simvastatin-equivalent daily dose of >42 mg (≥15,561 mg per year) significantly reduced PLC risk, even after adjusting for incident cirrhosis. These findings inform optimal statin use for PLC prevention and suggest avenues for future research.

We quantified cumulative statin exposure during follow-up by standardizing doses based on LDL-lowering intensity and computing annual exposure as simvastatin-equivalent mg/year. Unlike most prior studies, which treated statin use as binary, or numeric, but solely on duration or used drug-specific recommended daily doses as units [44,45,46,47,48,49], our approach accounts for biological effect. Recommended doses reflect not only potency but also factors like bioavailability and formulation, making them more suitable for drug utilization studies than for evaluating chemoprevention. Our analysis showed that a simvastatin-equivalent daily dose > 40 mg was effective for PLC prevention in the fully adjusted model, including incident cirrhosis. This differs from our cirrhosis analysis [13], where a simvastatin-equivalent daily dose of ≥20 mg (or >40 mg for those <54 years) was protective. Notably, statin duration alone—without dose consideration—did not show a dose-dependent effect. These findings suggest that high-intensity statin therapy [50] is necessary for effective PLC prevention, regardless of formulation.

One of our objectives was to assess whether statin-associated PLC protection varies by sex, age, or metabolic syndrome, a proxy for insulin resistance, which promotes premature liver senescence by hyperinsulinemia [34] and may alter statin response. Statins are known to modulate the hepatic transcriptome in a sex-specific manner in animal models [12], suggesting potential sex-related disparities. However, consistent with our cirrhosis analysis, we found no significant sex interaction, though the study may have been underpowered to detect it. In contrast to the age-dependent protection observed in our cirrhosis analysis [13], where older individuals benefited more, no clear age-related trend emerged for PLC. Similarly, metabolic syndrome did not modify the statin effect, with or without age interaction. Cirrhotic status also did not show effect modification (Supplemental Table S7). While further validation is needed, these findings suggest that statin protection against PLC may not be strongly influenced by sex, age, insulin resistance or disease stage. This has important implications for individualized chemoprevention strategies. Despite known age- and sex-related differences in statin safety [51], the efficacy of statins in PLC prevention appears consistent across these subgroups. The hazard ratios for baseline statin use in HCC and CCA were similarly protective, suggesting that statins may also confer benefit against CCA. Further investigation across disciplines is warranted.

In the present study, our primary objective was to evaluate the association between statin exposure and the risk of PLC. For etiologic analyses addressing exposure–outcome relationships, estimation of cause-specific hazards using the Cox PH model is generally recommended, as it quantifies the relative hazard of the event of interest among individuals who remain at risk. Competing risk approaches, such as the Fine–Gray model, estimate subdistribution hazards that primarily reflect differences in cumulative incidence and are more commonly used for risk prediction or prognosis. The subdistribution method fails to isolate distinct causal effects on the competing risk. A factor that increases the cause-specific risk of one outcome may decrease the subdistribution of another or respectively eliminate the probability that the event will occur [52,53]. Subdistribution hazard ratios cannot be interpreted as having a direct effect on the instantaneous risk of the event among those still at risk. Accordingly, we considered the cause-specific Cox model to be the most appropriate primary analytic approach.

Statin exposure status was continuously updated throughout follow-up in our study. Accordingly, treatment classification reflected the temporal sequence between medication exposure and development of primary liver cancer (PLC). Together, these design features helped ensure appropriate temporal ordering of exposure and outcome while minimizing the potential for immortal time bias.

Our study has limitations. Statin use was determined based on prescription records from both inpatient and outpatient services, without data on patient adherence. Statin exposure was standardized using dose equivalence based on LDL-cholesterol-lowering potency. As a result, potential differences in biological effects beyond LDL lowering (e.g., pleiotropic or anti-inflammatory effects), particularly when switching between statin formulations, may not be fully captured in this analysis. Smoking, another risk factor for PLC development [54], was not included in our analysis owing to the lack of time-specific data on smoking history. The VA population is predominantly male (~90%), limiting the statistical power to conclude sex difference and the generalizability of our findings to the general population. The use of ICD-9 and ICD-10 codes to define PLC incidence and cancer-type-specific incidence may have led to misclassification. The transition between coding systems during the study period could introduce minor differences in diagnostic classification, but any potential misclassification would likely be nondifferential with respect to statin exposure and is unlikely to materially bias the observed associations.

As our dataset was limited to VA EHR records, any care received outside the VA system may have been missing. As with all observational studies using real-world data, residual confounding cannot be fully excluded given constraints in routinely collected clinical variables. Cohort definition was based on a validated EHR algorithm to identify NAFLD in longitudinal datasets [16], incorporating chronic liver enzyme elevation along with clinical diagnoses related to metabolic dysfunction. This approach may have excluded individuals with steatotic liver disease and persistently normal liver enzymes. Accordingly, our findings are most applicable to individuals with chronic liver injury (elevated liver enzymes) linked to metabolic dysfunction. Given heterogeneity in pathophysiology across chronic liver diseases, these results may not be generalizable to other etiologies, including viral hepatitis, alcoholic liver disease, or Metabolic-dysfunction-associated Alcohol-related Liver Disease (MetALD). Finally, the potential hepatoprotective effects of statins on PLC risk may be modified by pharmacogenomic variation or concomitant medications/dietary supplements (e.g., fenofibrates, ezetimibe, theophylline) [55,56,57,58,59], which were not evaluated in this study. These factors warrant further investigation.

## 5. Conclusions

Our study suggests that statins may offer a dose-dependent protective effect against primary liver cancer (PLC), with high-intensity therapy (simvastatin-equivalent > 40 mg daily) associated with substantial benefit, independent of age, insulin resistance, or disease stage. In the absence of evidence from randomized clinical trials, further validation in independent cohorts, particularly those with a higher proportion of women, as well as studies using clinical trial simulation approaches, are warranted.

## Figures and Tables

**Table 1:** Baseline clinical characteristics of the study population.

Baseline Clinical Characteristics		Statin Use	
	Overall	No (34%)	Yes (66%)
**Demographics**			
Age *, median [IQR]	62 [53, 68]	57 [46, 65]	63 [56, 69]
Male sex *, %	91.6	88.6	93.2
Race *, % White/Black or African American/Asian/Am. Indian or Alaska Native/ Native Hawaiian or Other Pacific Islander/Unknown	78.0/11.2/0.9/0.7/0.9/8.3	75.0/12.5/1.0/0.8/0.9/9.8	79.6/10.5/0.9/0.6/0.9/7.5
Ethnicity *, % Hispanic or Latino/Non-Hispanic or Latino/Unknown	6.0/89.8/4.2	6.9/87.8/5.3	5.5/90.8/3.7
BMI *, kg/m^2^	31.60 [28.20, 35.60]	31.50 [28.10, 35.40]	31.60 [28.30, 35.70]
**Cardiometabolic features**			
Metabolic syndrome (3+ metabolic factors) *, %	78.6	68.4	83.9
Obesity *, %	62.6	61.9	63.0
Diabetes mellitus *, %	39.8	22.3	48.8
Hypertension *, %	73.9	58.7	81.8
Hyperlipidemia *, %	79.5	49.9	94.8
Coronary artery disease *, %	24.4	8.9	32.5
**Hepatic fibrosis/cirrhosis**			
FIB-4 score *, median [IQR]	1.26 [0.88, 1.80]	1.12 [0.77, 1.66]	1.33 [0.95, 1.86]
Clinical diagnosis of cirrhosis *, %	0.5	0.6	0.4
**Comedication use, %**			
Metformin *	26.7	12.7	34.0
ACE inhibitors/angiotensin receptor blockers *	58.0	36.0	69.4
Beta-blockers *	38.2	21.4	46.9
Aspirin/non-aspirin NSAIDs *	54.8	49.4	57.5
Vitamin D supplements *	9.6	8.3	10.2

* *p* < 0.05 for comparisons between statin users and non-users, using the Mann–Whitney U test for continuous variables or the χ^2^ test for categorical variables.

**Table 2:** Cumulative incidence of PLC in the overall population, stratified by sex and age group.

	Cirrhosis Dx at Baseline			No Cirrhosis Dx at Baseline		
	No of Events	No of Persons at Risk	Cumulative Incidence with 95% CI	No of Events	No of Persons at Risk	Cumulative Incidence with 95% CI
Overall	134	1556	8.6% [7.3, 10.1]	2583	328,021	0.8% [0.76, 0.82]
Women	6	91	6.6% [3.1, 13.7]	77	27,496	0.3% [0.22, 0.35]
Women ≤ 65 yo	3	67	4.5% [1.5, 12.4]	62	23,655	0.3% [0.20, 0.34]
Women > 65 yo	3	24	12.5% [4.3, 31.0]	15	3840	0.4% [0.24, 0.64]
Men	128	1465	8.7% [7.4, 10.3]	2506	300,525	0.8% [0.80, 0.87]
Men ≤ 65 yo	77	864	8.9% [7.2, 11.0]	1411	200,739	0.7% [0.67, 0.74]
Men > 65 yo	51	601	8.5% [6.5, 11.0]	1095	99,778	1.1% [1.03, 1.16]

PLC, primary liver cancer; Dx, diagnosis; CI, confidence interval; yo, years old.

**Table 3:** Association between incident PLC and statin use at baseline period in the overall population.

Baseline statin use	**Model 1**	**Model 2**	**Model 3**	**Model 4**	**Model 5**	**Model 6**
	1.20 [1.08, 1.33], 0.0004	0.88 [0.80, 0.98], 0.018	0.81 [0.73, 0.90], <0.0001	0.64 [0.57, 0.71], <0.0001	0.64 [0.58, 0.72], <0.0001	0.66 [0.59, 0.74], <0.0001

**Table 4:** The association between incident PLC and statin use during the 10-year follow-up in the overall population.

	Model 4	Model 6
	HR [95%CI], *p*-value	HR [95%CI], *p*-value
**Cumulative Statin Dose, mg/year**		
0	Ref	Ref
1–6960	1.10 [0.96–1.26], 0.183	1.13 [0.98–1.30], 0.085
6961–15,560	0.86 [0.74–0.99], 0.041	0.91 [0.78–1.05], 0.202
≥15,561	0.77 [0.66–0.90], 0.0008	0.84 [0.72–0.98], 0.024
**Cumulative Statin Duration, days/year**		
0	Ref	Ref
1–122	1.02 [0.83–1.24], 0.874	1.05 [0.86–1.29], 0.612
123–244	0.85 [0.70–1.03], 0.101	0.89 [0.74–1.08], 0.237
≥245	0.92 [0.81–1.05], 0.231	0.98 [0.86–1.12], 0.764

PLC, primary liver cancer; HR, hazard ratio; CI, confidence interval.

## Data Availability

The United States Department of VA imposes legal restrictions on access to Veterans’ healthcare data, which includes both identifiable and sensitive patient information. In accordance with VA privacy and data security policies, as well as regulatory requirements, the analytical datasets used in this study are not permitted to leave the VA firewall without an approved data use agreement. This limitation is consistent with other studies utilizing VA data. However, VA data are made freely available to researchers working within the VA firewall, provided they have an approved VA study protocol. For more information, please visit https://www.virec.research.va.gov or contact the VA Information Resource Center (VIReC) at vog.av@CeRIV.

## Supplementary Materials

The following supporting information can be downloaded at: https://www.mdpi.com/article/10.3390/cancers18071132/s1. Table S1: LDL-lowering intensity scores used to compute simvastatin-equivalent doses (mg); Table S2. Missing Data Counts for Variables Included in the Analysis; Table S3: Frequency of specific statin formulations used at baseline; Table S4: Cancer type distribution and type-specific cumulative incidence; Table S5. Association between incident PLC and baseline statin use by age 65 years and sex; Table S6: Association between cancer type–specific events and baseline statin use; Supplemental material: Cancer-specific incidence assessment and statin’s protective effects on specific cancer types; Table S7: Interactions between statin exposure and age, metabolic syndrome, and cirrhosis status in time-dependent Cox proportional hazards models for incident primary liver cancer, adjusted for covariates including cirrhotic status (Model 6).
