# Supplementary material for: Association of Statin Use with Reduced Primary Liver Cancer Risk, Independent of Age and Cirrhosis Protection in MASLD
Source: Cancers (Basel). 2026 Apr 1;18(7):1132. doi: 10.3390/cancers18071132 (PMC13072028; doi:10.3390/cancers18071132)
Supplement: Supplementary file 1 [file cancers-18-01132-s001.zip › cancers-4175758-supplementary.pdf]

Supplemental tables

Supplemental Table 1: LDL-lowering intensity scores used to compute simvastatin-equivalent doses (mg)

	**Conversion ratio**
Rosuvastatin	8
Atorvastatin	2
Simvastatin	1
Pravastatin	0.5
Lovastatin	0.5
Fluvastatin	0.25

The conversion score was developed based on the published conversion table (Laufs U, Filipiak KJ, Gouni-Berthold I, Catapano AL, group Sew. Practical aspects in the management of statin-associated muscle symptoms (SAMS). Atheroscler Suppl 2017;26:45-55).

Supplemental Table 2. Missing Data Counts for Variables Included in the Analysis

Variables	N of missing data	% of missing data
Age_at_index	9	<0.1%
Age_Group	9	<0.1%
Male	0	0.0%
age_sex	9	<0.1%
Race	0	0.0%
ETHNICITY_SOURCE_VALUE	0	0.0%
BMI_Most_Recent_Baseline	30,070	9.1%
Serum_ALT_Baseline	26,839	8.1%
Serum_AST_Baseline	26,839	8.1%
Serum_ALP_Value_Baseline	18,794	5.7%
Bilirubin_Value_Baseline	31,701	9.6%
Serum_Albumin_Value_Baseline	41,988	12.7%
Platelet_Count_Baseline	26,839	8.1%
FIB4_Score_Baseline	26,839	8.1%
Diabetes_Baseline_Flag	0	0.0%
Hypertension_Baseline_Flag	0	0.0%
Hyperlipidemia_Flag_Baseline	0	0.0%
CAD_Flag_Baseline	0	0.0%
Statin_Baseline_Flag	0	0.0%
ATORVASTATIN_Baseline_Flag	0	0.0%
ROSUVASTATIN_Baseline_Flag	0	0.0%
SIMVASTATIN_Baseline_Flag	0	0.0%
PRAVASTATIN_Baseline_Flag	0	0.0%
FLUVASTATIN_Baseline_Flag	0	0.0%
Other_STATIN_Baseline_Flag	0	0.0%
Medication_Metformin_Flag_Baseline	0	0.0%
Medication_ACE_ARB_Flag_Baseline	0	0.0%
Medication_BB_Flag_Baseline	0	0.0%
Medication_NSAID_Asp_Flag_Baseline	0	0.0%
Vitamin_D_Flag_Baseline	0	0.0%

Supplemental Table 3: Frequency of specific statin formulations used at baseline

	**Baseline use, %**
Rosuvastatin	9.3%
Atorvastatin	2.6%
Simvastatin	57.1%
Pravastatin	6.6%
Fluvastatin	2.3%
Others	5.9%

Supplemental table 4: Cancer type distribution and type-specific cumulative incidence

	No of cases	Percentage, %	Cumulative incidence with 95% CI, %
**HCC only**	**1708**	**62.9%**	0.52% [0.49%, 0.54%]
**Any CCA**	**239**	**8.8%**	0.07% [0.06%, 0.08%]
IH-CCA only	127	4.7%	0.04% [0.03%, 0.05%]
Unclassified	91	3.4%	0.03% [0.02%, 0.03%]
Overlapping sites or co-coded as IH and 'unspecified'	21	0.8%	0.01% [0.00%, 0.01%]
**HCC and CCA**	**118**	**4.3%**	0.04% [0.03%, 0.04%]
**Unspecified**	**652**	**24.0%**	0.20% [0.18%, 0.21%]
Secondary liver cancer to be excluded	0	0%	

HCC, hepatocellular carcinoma; **CCA**, cholangiocarcinoma; **IH**, intrahepatic; **CI**, confidence interval. A total of 2,717 cases were further classified into mutually exclusive cancer-type categories. Among 118 cases co-diagnosed with HCC and CCA within 12 months, 91 were co-diagnosed with HCC and IH-CCA, of which 70 had HCC and IH-CCA only (without overlapping sites or unspecified types).

Supplemental Table 5. Association between incident PLC and baseline statin use by age 65 years and sex

	No of events	Persons at risk	Unadjusted	Model 1	Model 2
			HR [95% CI], p-value	HR [95% CI], p-value	HR [95% CI], p-value
**Women ≤ 65**	46	23,722	1.88 [1.02, 3.49], .04	1.40 [0.73, 2.68], .31	1.46 [0.76, 2.79], .26
**Women > 65**	15	3,864	0.64 [0.23, 1.79], .39	0.58 [0.20, 1.68], .32	0.61 [0.21, 1.80], .37
**Men ≤ 65**	905	201,603	1.29 [1.12, 1.49], .0004	0.85 [0.73, 0.98], .02	0.75 [0.65, 0.87], .0002
**Men > 65**	835	100,379	0.79 [0.68, 0.91], .0017	0.75 [0.65, 0.87], .0002	0.69 [0.59, 0.80], <.0001

HR, hazard ratio; **95% CI**, 95% confidence interval.

Model 1 was adjusted for age (continuous), race/ethnicity (non-Hispanic White, Hispanic, and Other), and baseline metabolic syndrome (yes or no).

Model 2 was adjusted for baseline cirrhosis (yes or no) in addition to the covariates included in Model 1.

Supplemental Table 6: Association between cancer type–specific events and baseline statin use

Individual model

	**No of events**	**HR and 95% CI, *P*-value**
**Cancer type**		
**HCC**	**1708**	**0.81 [0.71, 0.93], .002**
**CCA**	**239**	**0.83 [0.59, 1.18], .30**
IH-CCA	127	1.05 [0.63, 1.74], .86
**HCC and CCA**	**118**	**0.82 [0.52, 1.31], .41**
**Unspecified**	**652**	**0.89 [0.73, 1.08], .24**

Due to the limited sample size, the model was adjusted for age (continuous), race/ethnicity, baseline metabolic syndrome (yes or no), and baseline cirrhosis diagnosis (yes or no).

Supplemental Table 7: Interactions between statin exposure and age, metabolic syndrome, and cirrhosis status in time-dependent Cox proportional hazards models for incident primary liver cancer, adjusted for covariates including cirrhotic status (Model 6)

	***p*-value**
**Cumulative Statin Dose, mg/year**	
3-way interaction dose * age * metabolic syndrome	0.70
2-way interaction dose * age	0.80
2-way interaction dose * metabolic syndrome	0.82
2-way interaction dose * cirrhotic status	0.18
2-way interaction age * metabolic syndrome	0.88
**Cumulative Statin Duration, days/year**	
3-way interaction duration * age * metabolic syndrome	0.82
2-way interaction duration * age	0.66
2-way interaction duration * metabolic syndrome	0.92
2-way interaction duration * cirrhotic status	0.09
2-way interaction age * metabolic syndrome	0.94
